# AI-assisted analysis of early fluid dynamics following aflibercept 8 mg in treatment-naïve neovascular AMD

**DOI:** 10.1038/s41433-026-04319-1

**Published:** 2026-02-25

**Authors:** Daniele Veritti, Valentina Sarao, Asia Amelia Martin, Francesco Bonini, Paolo Lanzetta

**Affiliations:** 1 Department of Medicine-Ophthalmology, University of Udine, Udine, Italy; 2 Istituto Europeo di Microchirurgia Oculare - IEMO, Udine and Milan, Udine, Italy

**Keywords:** Macular degeneration, Retinal diseases

## Abstract

**Purpose:**

To evaluate early morphological changes following intravitreal aflibercept 8 mg in treatment-naïve neovascular age-related macular degeneration (nAMD) using artificial intelligence (AI)-assisted optical coherence tomography (OCT) segmentation.

**Methods:**

Thirty eyes with treatment-naïve subfoveal nAMD received three monthly aflibercept 8 mg injections. OCT scans were obtained at baseline and days 1, 7, 14, 30, 60, and 90. AI-based segmentation software (Fluid Monitor, RetInSight GmbH) quantified intraretinal fluid (IRF), subretinal fluid (SRF), and pigment epithelial detachment (PED) volumes in the central 6-mm area. Morphologic changes were correlated with best-corrected visual acuity (BCVA).

**Results:**

IRF, SRF, and PED showed distinct reduction patterns. In eyes with IRF (*n* = 22), mean volume decreased by 65% at day 1, 94% at day 14, and 96% at day 90 (*p* < 0.05). Among eyes with SRF (*n* = 28), SRF volume decreased by 18%, 84%, and 99% at days 1, 14, and 90, respectively (*p* ≤ 0.002). PED volume declined more gradually among affected eyes (*n* = 23), with mean reductions of 7% at day 1, 34% by day 14, and 53% by day 90 (*p* ≤ 0.023). BCVA improved from 51.8 ± 14.4 to 62.6 ± 14.4 ETDRS letters by day 90 (mean gain + 10.8; *p* < 0.001). Multivariate regression identified a significant association between IRF reduction at day 14 and BCVA gain at day 90 (*β* = −0.018, *p* = 0.036).

**Conclusions:**

Aflibercept 8 mg induced rapid, compartment-specific morphologic responses in treatment-naïve nAMD, with early IRF resolution and significant BCVA improvement. AI-assisted analysis provided detailed pharmacodynamic insights and may support individualised treatment strategies during the loading phase.

## Introduction

Neovascular age-related macular degeneration (nAMD) is a leading cause of severe, irreversible visual loss and poses an increasing public health burden as populations age [1]. Anti-vascular endothelial growth factor (anti-VEGF) therapy has revolutionised the treatment of nAMD, significantly improving visual and anatomical outcomes compared to the natural history of the disease [2, 3]. Aflibercept, a recombinant fusion protein targeting VEGF-A, VEGF-B, placental growth factor and galectin-1, has been widely used in clinical practice at the standard dose of 2 mg [4–6]. Recently, an 8 mg high-dose formulation was introduced to sustain VEGF suppression and reduce treatment burden while maintaining efficacy [7–9]. The PULSAR trial demonstrated that aflibercept 8 mg administered every 12 or 16 weeks was non-inferior to 2 mg every 8 weeks in terms of best-corrected visual acuity (BCVA), with promising anatomical outcomes and an excellent safety profile [10]. While the efficacy and durability of aflibercept 8 mg are increasingly supported by evidence from randomised trials and real-world reports, current literature has primarily focused on long-term functional outcomes and dosing intervals [11–18]. In contrast, the early morphological dynamics induced by high-dose aflibercept remain largely uncharacterised. A detailed understanding of fluid resolution kinetics within the first days and weeks post-injection is crucial to elucidate the pharmacodynamic behaviour of the drug, optimise loading-phase regimens and retreatment intervals, and potentially predict long-term efficacy.

AI-assisted OCT segmentation enhances detection and quantification of fluid compartments, enabling reproducible assessment of treatment response [19–23].

In this prospective study, we aimed to characterise and quantify the early temporal changes in retinal morphology following treatment with aflibercept 8 mg in patients with treatment-naïve nAMD, using AI-assisted analysis of spectral-domain optical coherence tomography (SD-OCT) images. We also investigated the association between anatomical changes and early functional outcomes. This analysis seeks to improve the understanding of the short-term morphologic effects of aflibercept 8 mg and to contribute to the optimisation of initial treatment strategies in clinical practice.

## METHODS

### Study design and participants

This prospective study was conducted at the Department of Medicine–Ophthalmology, University of Udine, between August 2024 and March 2025. The study protocol adhered to the tenets of the Declaration of Helsinki and received approval from the institutional review board.

Eligible patients included adults aged ≥ 50 years with treatment-naive nAMD with active subfoveal macular neovascularisation (MNV), confirmed by multimodal imaging.

Exclusion criteria included previous treatment for nAMD in the study eye, any other ocular condition affecting visual acuity, history of intraocular surgery within 3 months, active ocular inflammation, and uncontrolled glaucoma. When both eyes of the same patient met the inclusion criteria, the eye with the better baseline BCVA was selected for analysis.

The presence and classification of MNV were confirmed by two independent retina specialists (DV and VS) based on multimodal imaging findings. MNV was classified according to consensus nomenclature as type 1, type 2, mixed type 1/2, type 3 neovascularisation, or polypoidal choroidal vasculopathy (PCV). Pigment epithelium detachments (PEDs) were classified based on internal reflectivity patterns into the following subtypes: predominantly serous PEDs, characterised by ≥ 50% serous fluid content and low internal reflectivity; purely fibrovascular PEDs, showing peaked elevations with uniformly moderate reflectivity; and predominantly fibrovascular PEDs, containing ≥ 50% fibrovascular tissue. In cases of uncertain classification, conventional angiography was used to determine the predominant PED component. The sample size of 30 eyes was chosen to provide exploratory, high-resolution data on fluid dynamics in the early post-treatment phase, enabling hypothesis generation for future larger studies.

### Treatment protocol and assessment schedule

All patients received intravitreal aflibercept 8 mg (0.07 mL of 114.3 mg/mL solution) at baseline (day 0), and subsequently at day 30, and day 60, as per label. Ophthalmic examinations were performed at baseline and days 1, 7, 14, 30, 60, and 90 following the initial treatment. Each examination protocol consisted of BCVA assessment using Early Treatment Diabetic Retinopathy Study (ETDRS) charts, a thorough ophthalmic examination comprising slit-lamp biomicroscopy and dilated fundus examination, and SD-OCT imaging (Spectralis, Heidelberg Engineering, Germany) utilising a dense map program 20° x 20° with high-speed mode. Results were expressed as mean ± standard deviation. At baseline and at the end of follow-up, additional diagnostic imaging was conducted, including fluorescein angiography (FA) and indocyanine green angiography (ICGA).

### AI-assisted fluid segmentation

Retinal fluid segmentation was performed using the Fluid Monitor software (RetInSight GmbH, Vienna, Austria), a validated AI-driven platform specifically designed for the automated identification and quantification of intraretinal fluid (IRF), subretinal fluid (SRF), and PED volumes on SD-OCT images. The system is powered by a deep convolutional neural network trained on a diverse dataset of expertly annotated, pathology-confirmed OCT scans [24, 25]. For each B-scan, the algorithm performs pixel-level classification to accurately distinguish pathological fluid compartments from normal retinal tissue. Quantitative metrics generated by the software include volumetric data for IRF, SRF, and PED within the central 6-mm macular area. The algorithm has been extensively validated across multiple clinical cohorts and reading centres, showing excellent agreement with manual grading (Pearson correlation coefficient *r* = 0.908), as well as high sensitivity in detecting fluid-related retinal structural changes [24, 25]. Despite the high level of automation, all segmentation results underwent manual verification by an experienced grader (VS), who was masked to the temporal sequence of the scans. No manual corrections of the automated segmentation were required.

### Statistical analysis

Statistical analyses were performed to assess longitudinal changes in BCVA and retinal fluid characteristics over the study period. Complete fluid resolution was defined as a residual fluid volume ≤ 5 nL within the central 6-mm macular area for each fluid compartment (IRF, SRF, and PED). Given the non-normal distribution of the data, non-parametric tests were applied. The Friedman test was used for repeated measures to evaluate intra-subject changes across timepoints, while Spearman’s rank correlation coefficient was employed to explore associations between morphological and functional parameters. Longitudinal changes across time points were assessed using repeated-measures models. When post hoc pairwise comparisons were performed, adjustments for multiple comparisons were applied to control the family-wise error rate. For analyses using change-from-baseline outcomes, baseline values were incorporated in the definition of the outcome variable and were not included as additional covariates. Descriptive statistics were used to summarise baseline demographic and clinical characteristics, as well as treatment outcomes. To examine the predictive value of fluid dynamics on visual function, two multivariable linear regression models were used: one tested the independent contribution of PED, SRF, and IRF volumes on BCVA, the other the effect of their changes on BCVA changes. Statistical significance was defined as a two-sided *p*-value of ≤ 0.05. A post hoc power analysis was performed to estimate the minimum detectable effect size for within-eye changes in OCT-derived fluid volumes. Assuming a two-sided α of 0.05 and 80% power, a sample size of 30 eyes allows detection of a standardised mean difference of approximately 0.5.

## RESULTS

### Patient’s characteristics

A total of 30 treatment-naive patients (30 eyes) with nAMD were enrolled (Table 1). The mean age was 74.6 ± 8.3 years, with female predominance (67%). Based on multimodal imaging, 16 patients (53.3%) presented with type 1 MNV, 7 patients (23.3%) with type 2 MNV, 5 patients (16.7%) with mixed type 1/2 MNV, and 2 patients (6.7%) with type 3 MNV. No cases of PCV were identified. Mean baseline BCVA was 51.8 ± 14.4 letters (approximate Snellen equivalent of 20/80). Mean baseline central retinal thickness (CRT) was 415.3 ± 125.0 μm. At baseline, 22 eyes (73.3%) exhibited IRF, 28 eyes (93.3%) had SRF, and 23 eyes (76.7%) presented with PED. Among these, 9 (39.1%) were classified as purely fibrovascular, 10 (43.5%) as predominantly fibrovascular, and 4 (17.4%) as predominantly serous. All patients completed the three initial loading doses of intravitreal aflibercept 8 mg and attended all scheduled visits through the 90-day follow-up.

**Table 1:** Baseline characteristics.

Characteristic	Value
Age, (years ± SD)	74.6 ± 8.3 (range 65–89)
Sex, *n* (%)	Female: 20 (66.7%)
Baseline BCVA, (ETDRS letters, mean ± SD)	51.8 ± 14.4 (approximate Snellen 20/80)
Baseline CRT, (µm, mean ± SD)	415.3 ± 125.0
MNV subtype, *n* (%)	Type 1: 16 (53.3%) Type 2: 7 (23.3%) Mixed type 1/2: 5 (16.7%) Type 3: 2 (6.7%)
Presence of fluid at baseline, *n* (%)	Intraretinal fluid: 22 (73.3%) Subretinal fluid: 28 (93.3%) Pigment epithelium detachment: 23 (76.7%)
Systemic comorbidities, *n* (%)	Hypertension: 14 (46.7%) Diabetes mellitus: 7 (23.3%) Dyslipidaemia: 10 (33.3%) Cardiovascular disease: 4 (13.3%)
Smoking status, *n* (%)	Current: 6 (20.0%) Former: 10 (33.3%) Never: 14 (46.7%)
Lens status, *n* (%)	Phakic: 16 (53.3%) Pseudophakic: 14 (46.7%)
Intraocular pressure, (mmHg, mean ± SD)	15.2 ± 2.8

*BCVA* best corrected visual acuity, *CRT* central retinal thickness, *ETDRS* Early Treatment Diabetic Retinopathy Study, *n* number, *SD* standard deviation.

### Visual acuity outcomes

**Fig. 1:**
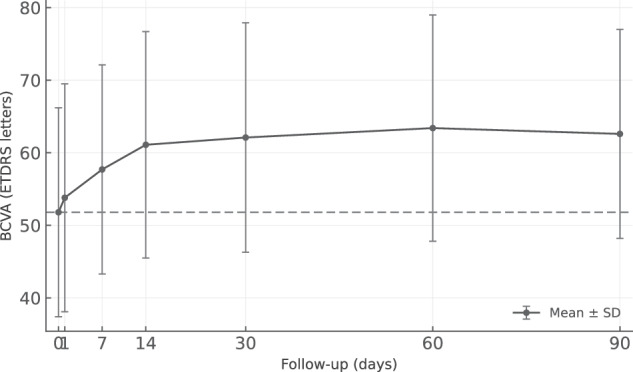
Mean best-corrected visual acuity (BCVA, ETDRS letters) over the loading phase with intravitreal aflibercept 8 mg in treatment-naïve nAMD eyes. Values represent mean ± standard deviation at baseline and days 1, 7, 14, 30, 60, and 90. A rapid and significant improvement was observed as early as day 1, with a mean gain of + 10.8 letters at day 90 (*p* < 0.001).

BCVA improved significantly from 51.8 ± 14.4 letters at baseline to 53.8 ± 15.7 letters at day 1 (*p* = 0.041), 57.7 ± 14.4 letters at day 7 (*p* < 0.001), and 62.6 ± 14.4 letters at day 90 (*p* < 0.001), representing a mean gain of + 10.8 letters (Fig. 1).

Visual improvement differed across MNV subtypes. Type 2 MNV showed the most rapid initial response, with a mean gain of 9.0 ± 5.1 letters at day 7, compared to 5.3 ± 5.3 letters for type 1 MNV. Mixed type 1/2 MNV demonstrated a more limited improvement (2.0 ± 1.0 letters at day 7), while type 3 MNV cases showed an intermediate early response (7.5 ± 0.7 letters). By day 90, mean letter gains were 10.4 ± 7.3 for type 1 MNV, 12.5 ± 6.9 for type 2 MNV, 5.3 ± 15.9 for mixed cases, and 11.5 ± 1.4 for type 3 MNV. Multivariate regression showed that IRF volume change at day 14 was independently associated with BCVA gain at day 90 (*β* = −0.018, 95% CI: −0.034 to −0.002, *p* = 0.036), whereas SRF and PED volume changes were not significant predictors (day 14 SRF change *β* = −0.0145, 95% CI: −0.033 to 0.004, *p* = 0.11; day 14 PED volume change *β* = −0.012, 95% CI: −0.028 to 0.004, *p* = 0.13). BCVA improvement was moderately correlated with IRF volume reduction (Spearman r = −0.41, *p* = 0.048), supporting the role of IRF as a potential biomarker of early functional response.

### Retinal morphological changes and fluid dynamics

**Fig. 2:**
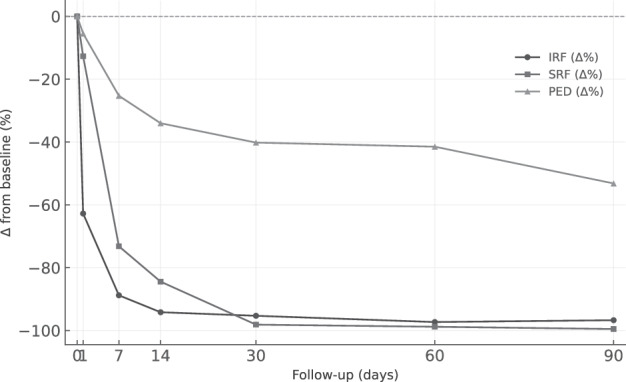
Percentage reduction from baseline in intraretinal fluid (IRF), subretinal fluid (SRF), and pigment epithelial detachment (PED) volumes over time. IRF is represented by the black line with circles, SRF by the dark grey line with squares, and PED by the light grey line with triangles.

The AI-assisted segmentation algorithm revealed distinct temporal patterns in the resolution of retinal fluid across different compartments (Table 2, Fig. 2).

**Table 2:** Functional and anatomical parameters over time.

Parameter	Baseline	Day 1	Day 7	Day 14	Day 30	Day 60	Day 90
BCVA (letters, mean ± SD)	51.8 ± 14.4	53.8 ± 15.7	57.7 ± 14.4	61.1 ± 15.6	62.1 ± 15.8	63.4 ± 15.6	62.6 ± 14.4
ΔBCVA (letters)	–	+2.0	+5.9	+9.3	+10.3	+11.6	+10.8
CRT (µm, mean ± SD)	415.3 ± 125.0	371.8 ± 101.5	311.2 ± 91.7	295.2 ± 83.7	278.4 ± 74.8	273.2 ± 60.3	265.4 ± 61.5
ΔCRT (µm)	–	−43.5	−104.1	−120.1	−136.9	−142.1	−149.9
ΔCRT (%)	–	−10.5%	−25.1%	−28.9%	−33.0%	−34.2%	−36.1%
IRF (nL, mean ± SD)	185.6 ± 377.0	69.1 ± 136.4	20.8 ± 50.7	10.8 ± 35.0	8.7 ± 26.0	5.0 ± 12.6	6.1 ± 15.3
IRF (nL, median (IQR))	19.1 (0.1–206.8)	1.9 (0.2–61.7)	1.9 (0.0–5.9)	0.0 (0.0–0.8)	0.1 (0.0–0.2)	0.0 (0.0–0.6)	0.0 (0.0–0.4)
ΔIRF (nL)	–	−116.5	−164.8	−174.8	−176.9	−180.6	−179.5
ΔIRF (%)	–	−65.0%	−88.8%	−94.2%	−95.3%	−97.3%	−96.8%
SRF (nL, mean ± SD)	251.3 ± 276.6	219.4 ± 289.1	67.4 ± 86.2	39.1 ± 61.4	4.7 ± 8.3	3.0 ± 6.0	1.2 ± 1.5
SRF (nL, median (IQR))	177.8 (16.3–372.4)	72.4 (7.8–208.1)	27.6 (3.4–106.9)	5.0 (0.7–52.9)	1.9 (0.1–3.8)	0.6 (0.0–2.8)	0.5 (0.0–2.6)
ΔSRF (nL)	–	−31.9	−183.9	−212.2	−246.6	−248.3	−250.1
ΔSRF (%)	–	−18.5%	−73.2%	−84.5%	−98.1%	−98.8%	−99.5%
PED (nL, mean ± SD)	490.0 ± 423.7	463.2 ± 382.8	366.1 ± 313.7	323.3 ± 249.0	293.1 ± 239.2	286.7 ± 220.4	229.4 ± 186.1
PED (nL, median (IQR))	381.2 (177.0–743.5)	380.1 (133.4–711.8)	339.2 (153.2–539.5)	277.5 (137.9–571.1)	291.7 (99.7–460.3)	336.7 (79.4–456.6)	229.0 (55.8–370.3)
ΔPED (nL)	–	−26.8	−123.9	−166.7	−196.9	−203.3	−260.6
ΔPED (%)	–	−7.3%	−25.3%	−34.0%	−40.2%	−41.5%	−53.2%

*BCVA* best corrected visual acuity, *CRT* central retinal thickness, *IQR* interquartile range, *IRF* intraretinal fluid, *n* number, *PED* pigment epithelium detachment, *SD* standard deviation, *SRF* subretinal fluid, Δ: change.

### Central retinal thickness

**Fig. 3:**
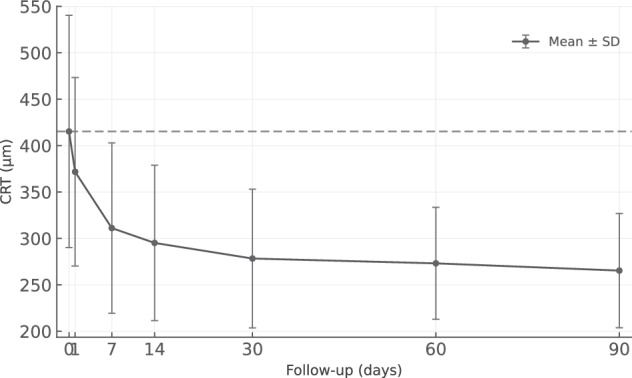
Temporal changes in central retinal thickness (CRT, µm) following intravitreal aflibercept 8 mg. Mean ± standard deviation values are shown at baseline and subsequent follow-up visits through day 90. CRT decreased significantly from baseline (415 µm) to day 90 (265 µm), corresponding to a 36.1% reduction (p < 0.0001).

Mean CRT decreased significantly from baseline (415.3 ± 125.0 μm) as early as day 1 (371.8 ± 101.5 μm, *p* = 0.0019) and continued to improve throughout the study period (Fig. 3). By day 7, mean CRT reduced to 311.2 ± 91.7 μm (*p* = 0.0011), representing a 25.1% reduction. By day 90, CRT reached 265.4 ± 61.5 μm (*p* < 0.0001), a total reduction of 36.1% (Fig. 3).

Between MNV subtypes, type 2 MNV showed the greatest CRT reduction (264.8 μm, 47.1% reduction) compared to type 1 MNV (85.1 μm, 23.3% reduction) at day 90. Mixed type 1/2 MNV showed a reduction of 202.3 µm (39.2%), while type 3 MNV exhibited a reduction of 182.0 µm (35.2%). These differences did not reach statistical significance.

### Intraretinal fluid

IRF demonstrated the most rapid response to treatment. Mean IRF volume at baseline was 186 ± 377 nL, decreasing by 65.0% at day 1 (69 ± 136 nL, *p* = 0.041), 88.7% at day 7 (21 ± 51 nL, *p* = 0.032), and 94.1% at day 14 (11 ± 35 nL, *p* = 0.047). By day 90, mean IRF volume reached 6 ± 15 nL, representing a 96.8% reduction (*p* = 0.038). The rate of IRF resolution was similar across MNV subtypes. At day 7, mean reductions were 81.0% for type 1 MNV, 86.0% for type 2 MNV, 79.6% for mixed type 1/2 MNV, and 79.7% for type 3 MNV (*p* = 0.27). By day 30, IRF volume reduction reached 93.4% in type 1 MNV, 97.6% in type 2 MNV, 92.3% in mixed type 1/2 MNV, and 95.0% in type 3 MNV (*p* = 0.34). Complete resolution of IRF was observed in 10 eyes (33.3%) at day 7, 18 eyes (60.0%) at day 14, and 27 eyes (90.0%) at day 90. The mean time to complete resolution was 27.3 ± 12.5 days for type 1, 30.0 ± 13.1 days for type 2, 20.4 ± 9.3 days for mixed type 1/2, and 22.1 ± 10.2 days for type 3 (*p* = 0.18).

### Subretinal fluid

SRF demonstrated a robust response to treatment. Mean SRF volume at baseline was 251.3 ± 276.6 nL, decreasing by 18.5% at day 1 (219.4 ± 289.1 nL, *p* = 0.022), 73.2% at day 7 (67.4 ± 86.2 nL, *p* = 0.002), and 84.5% at day 14 (39.1 ± 61.4 nL, *p* = 0.002). By day 90, mean SRF volume reached 1.2 ± 1.5 nL, corresponding to a 99.5% reduction (*p* = 0.001).

The rate of SRF resolution varied across MNV subtypes. At day 7, mean reductions were 78.0% for type 1 MNV, 77.0% for type 2 MNV, 74.0% for mixed type 1/2 MNV, and 25.0% for type 3 MNV. By day 30, reductions reached 90.7% in type 1, 98.1% in type 2, 88.1% in mixed type 1/2, and 92.6% in type 3 (*p* = 0.31). Complete resolution was achieved in 8 eyes (26.7%) at day 7, 15 eyes (50.0%) at day 14, and 30 eyes (100%) at day 90. The mean time to resolution was 37.5 ± 27.9 days for type 1, 23.0 ± 12.1 days for type 2, 40.4 ± 11.3 days for mixed type 1/2, and 75.0 ± 21.2 days for type 3 (*p* = 0.46).

### Pigment epithelial detachment

PED volume showed a more gradual response. Mean baseline PED volume was 490.0 ± 423.7 nL, with a 7.3% reduction at day 1 (463.2 ± 382.8 nL, *p* = 0.110), 25.3% at day 7 (366.1 ± 313.7 nL, *p* = 0.011), and 34.0% at day 14 (323.3 ± 249.0 nL, *p* = 0.023). By day 30, mean PED volume had decreased by 40.2% (293.1 ± 239.2 nL, *p* = 0.016), and by day 90 the reduction reached 53.2% (229.4 ± 186.1 nL, *p* = 0.007). PED response was subtype-specific. At day 7, predominantly serous PEDs showed the greatest reduction in volume (31.5%), while predominantly fibrovascular PEDs demonstrated a more modest decline (22.8%), and purely fibrovascular PEDs exhibited the smallest reduction (17.4%, *p* = 0.028). By day 90, serous PEDs achieved a 72.6% volume reduction, compared to 56.3% for predominantly fibrovascular and 48.7% for purely fibrovascular PEDs (*p* = 0.041).

### Fluid-free status

The proportion of eyes achieving a completely fluid-free macula increased progressively. By day 7, 8 eyes (26.7%) were fluid-free, rising to 15 eyes (50.0%) at day 14, 20 eyes (66.7%) at day 30, 25 eyes (83.3%) at day 60, and 27 eyes (90.0%) at day 90.

### Safety outcomes

No serious ocular or systemic adverse events were reported. Minor adverse events included transient conjunctival hyperaemia in 4 patients (13.3%), punctate keratopathy in 2 patients (6.7%), and subconjunctival haemorrhage in 3 patients (10.0%), all of which resolved without sequelae. No cases of intraocular inflammation or retinal pigment tears were observed.

## DISCUSSION

In this prospective observational study, we investigated the early morphological changes occurring in treatment-naïve nAMD patients following intravitreal administration of aflibercept 8 mg. Using AI-assisted segmentation and quantification of fluid compartments, we observed early, compartment-specific anatomical improvements, with distinct resolution dynamics based on fluid type and MNV subtype. These changes were accompanied by early and sustained visual acuity gains, observed as soon as one day after injection and persisting through the 90-day follow-up.

Unlike previous studies assessing fluid response at monthly intervals, our high-frequency imaging protocol allowed for high-definition temporal resolution, capturing morphological changes as early as 24 h post-injection. The sequence of fluid resolution followed a consistent pattern—IRF showed the fastest decline (65% at day 1, 94% at day 14), followed by SRF and then PED. The prompt therapeutic effect observed aligns with aflibercept’s pharmacodynamic profile. Our supporting mathematical model indicates that an 8 mg dose maintains VEGF suppression below biological thresholds for extended periods, providing a pharmacokinetic rationale for both rapid onset and durability of action [8, 9].

In our cohort, early anatomical responses to aflibercept 8 mg were broadly similar between type 1 and type 2 MNV. At day 7, IRF reduction was slightly greater in type 2 lesions (86.0% vs 81.0% for type 1), while SRF resolution was nearly identical (77.0% vs 78.0%). Mixed type 1/2 lesions showed a more limited functional response ( + 2.0 letters at day 7) but still achieved substantial anatomical improvement, with IRF and SRF reductions of 79.6% and 74.0%, respectively, by day 7. Interestingly, type 3 lesions demonstrated an intermediate early functional gain ( + 7.5 letters at day 7) but a comparatively delayed resolution of SRF (25.0% reduction at day 7, improving to > 90% by day 30). This may reflect the distinct pathophysiology of type 3 neovascularisation.

PED showed the slowest resolution and highest variability. Serous PEDs responded more rapidly than fibrovascular PEDs, consistent with their simpler fluid-filled structure, while fibrovascular PEDs, often associated with type 1 MNV, showed modest reductions due to their complex, tissue-rich architecture. Given the limited number of eyes in each PED subgroup, these differences should be interpreted as exploratory observations.

The fluid resolution kinetics observed with aflibercept 8 mg in our study can be contextualised with those recently reported with faricimab in a similar AI-assisted analysis of nAMD patients [20]. In that study, conducted with the same segmentation platform and a nearly identical follow-up schedule, faricimab (6 mg) led to a 51% reduction in PED volume by day 14, compared to 34.0% with aflibercept 8 mg in our cohort. IRF and SRF resolution, however, appeared comparable, with both agents achieving over 80% fluid reduction by the two-week mark. Although no head-to-head conclusions can be drawn, these findings suggest that faricimab may show a distinct early pattern of PED modulation, potentially related to its dual inhibition of VEGF-A and Ang-2. In contrast, aflibercept 8 mg showed similarly rapid reductions in IRF and SRF, and a particularly high rate (90%) of complete IRF clearance by day 90, highlighting its marked activity in resolving intraretinal exudation. These differences may reflect distinct pharmacologic profiles and mechanisms of action, and support the rationale for personalised therapeutic strategies based on fluid composition and neovascular subtype.

The proportion of eyes achieving a fluid-free macula increased steadily, reaching 50.0% at Day 14 and 90.0% at Day 90. This is in line with recent reports from other agents. For instance, in the comparable analysis of faricimab, complete fluid resolution was observed in 63% of eyes at Day 15 and 100% at Day 120. While such comparisons must be interpreted cautiously due to differences in study designs, patient populations, and assessment methodologies, our findings suggest that aflibercept 8 mg offers robust early anatomical and functional activity among next-generation anti-VEGF therapies [20].

The anatomical improvements in our study were accompanied by substantial visual gains, with mean BCVA improving from 51.8 letters at baseline to 62.6 letters at day 90 (a 10.8-letter gain). This early visual response appears more pronounced than that reported in phase 3 trials of aflibercept 8 mg. In the PULSAR trial, patients receiving aflibercept 8 mg every 12 or 16 weeks achieved mean BCVA gains of 6.7 and 6.2 letters, respectively, at week 48. This difference may reflect patient selection, tighter follow-up, or baseline characteristics, as commonly observed in small prospective cohorts [10].

Interestingly, the pattern of visual acuity improvement mirrored the anatomical response patterns across MNV subtypes. Type 2 MNV showed the most rapid initial visual gains ( + 9.0 letters at day 7), consistent with early fluid resolution. This correlation between anatomical and functional improvements supports the notion that early fluid resolution, particularly of IRF, may represent an exploratory biomarker associated with favourable visual outcomes [10, 11]. This highlights the potential role of early IRF clearance as a biomarker of long-term prognosis.

The complete resolution of IRF in 90% of eyes by day 90 is particularly noteworthy, as persistent IRF has been consistently associated with poorer visual outcomes in numerous studies [12, 13]. The rapid clearance of IRF observed with aflibercept 8 mg may therefore have important implications for long-term visual function. Similarly, the complete resolution of SRF in 100% of eyes by day 90 is encouraging, although the clinical significance of residual SRF remains debated, with some studies suggesting that persistent SRF may be less detrimental to visual outcomes than IRF [14, 15].

While IRF and SRF resolved rapidly, PED resolution remained incomplete, in line with findings from the CANDELA trial [13]. The clinical implications of residual PED—particularly fibrovascular types—remain to be clarified. Long-term data are needed to assess whether partial resolution predisposes to complications such as RPE tears or atrophy.

These fluid-specific and subtype-specific response patterns have important clinical implications for personalised care. For instance, patients with type 2 MNV and predominant IRF may be good candidates for extended treatment intervals following the loading phase, while those with fibrovascular PED and type 1 MNV may benefit from closer monitoring or shorter dosing cycles.

This study has several strengths. The use of AI-based volumetric analysis allowed objective, reproducible, and high-resolution assessment of treatment response. Our dense follow-up schedule enabled fine-grained temporal analysis often unavailable in standard clinical trials. Moreover, the exploratory stratification by MNV subtype and PED morphology adds depth to the understanding of variability in treatment response.

Nonetheless, some limitations must be acknowledged. First, the sample size was relatively modest, limiting statistical power, particularly for subgroup analyses. Although the sample size was sufficient to detect moderate-to-large within-eye effects, the study was not powered for confirmatory hypothesis testing or for stable multivariable prediction modelling. Accordingly, regression analyses should be considered exploratory and not intended to establish validated predictive models. Second, the follow-up period of 90 days captures only the loading phase response and does not address the long-term durability of the observed effects. Third, as an observational study without a control group, we cannot directly compare the efficacy of aflibercept 8 mg with standard-dose anti-VEGF agents or other treatment modalities. Selection bias may have also influenced results, as tertiary care patients may not represent the broader nAMD population. In addition, no cases of PCV were included in this cohort. Therefore, the present findings may not be generalisable to populations in which PCV is more prevalent, such as Asian or non-Caucasian cohorts.

The safety profile observed in our study was favourable, with no serious adverse events reported. This aligns with the results from the PULSAR trial [10]. However, our sample size is insufficient to detect rare adverse events, and longer-term surveillance in larger populations remains essential.

Future research should focus on several key areas. Longer-term studies are needed to assess the durability of the anatomical and functional improvements observed during the loading phase and to determine optimal treatment intervals for maintenance therapy. Comparative studies directly contrasting aflibercept 8 mg with other second-generation anti-VEGF agents would provide valuable insights into relative efficacy and safety profiles. Additionally, investigations into biomarkers predictive of response to high-dose aflibercept could facilitate more personalised treatment approaches. Finally, real-world studies evaluating the impact of aflibercept 8 mg on treatment burden, patient adherence, and healthcare resource utilisation would complement the efficacy data with important practical considerations.

In conclusion, our study demonstrates that aflibercept 8 mg induces rapid and substantial morphological improvements in treatment-naïve nAMD, with distinct temporal patterns of fluid resolution across different compartments and MNV subtypes. These anatomical changes are accompanied by significant early visual gains. The pronounced and accelerated fluid resolution observed with high-dose aflibercept may contribute to improved long-term outcomes and potentially support extended treatment intervals, thereby addressing the dual goals of optimising efficacy and reducing treatment burden. These findings provide valuable insights into the early pharmacodynamics of aflibercept 8 mg and may help inform treatment strategies in clinical practice.

## Summary

### What was known before:

- High-dose aflibercept (8 mg) extends treatment intervals while maintaining efficacy in nAMD.
- Early morphological responses during the loading phase, particularly assessed with AI, were not well characterised.

### What this study adds:

- AI-assisted OCT revealed rapid resolution of intraretinal and subretinal fluid after aflibercept 8 mg.
- Early IRF reduction at 2 weeks was associated with later visual improvement.
- AI-based fluid analysis provided novel pharmacodynamic insights to support individualised treatment strategies.

## Data Availability

The datasets generated during and/or analysed during the current study are available from the corresponding author on reasonable request.
